# Overview of Syntheses and Molecular-Design Strategies for Tetrazine-Based Fluorogenic Probes

**DOI:** 10.3390/molecules26071868

**Published:** 2021-03-26

**Authors:** Sang-Kee Choi, Jonghoon Kim, Eunha Kim

**Affiliations:** 1 Department of Molecular Science and Technology, Ajou University, Suwon 16499, Korea; huci42@ajou.ac.kr; 2 Department of Chemistry, Soongsil University, Seoul 06978, Korea

**Keywords:** tetrazine, click chemistry, fluorogenic, bioimaging

## Abstract

Various bioorthogonal chemistries have been used for fluorescent imaging owing to the advantageous reactions they employ. Recent advances in bioorthogonal chemistry have revolutionized labeling strategies for fluorescence imaging, with inverse electron demand Diels–Alder (iEDDA) reactions in particular attracting recent attention owing to their fast kinetics and excellent specificity. One of the most interesting features of the iEDDA labeling strategy is that tetrazine-functionalized dyes are known to act as fluorogenic probes. In this review, we will focus on the synthesis, molecular-design strategies, and bioimaging applications of tetrazine-functionalized fluorogenic probes. Traditional Pinner reaction and “Pinner-like” reactions for tetrazine synthesis are discussed here, as well as metal-catalyzed C–C bond formations with convenient tetrazine intermediates and the fabrication of tetrazine-conjugated fluorophores. In addition, four different quenching mechanisms for tetrazine-modified fluorophores are presented.

## 1. Introduction

Fluorescence imaging is one of the most important scientific tools for understanding biological systems [1]. To monitor the ongoing processes in innate biological systems, many different fluorescent bioprobes have been developed [2,3,4,5,6]. For successful fluorescence imaging, the preparation of fluorescent bioprobes must include minimal perturbation of the original properties of the biomolecule and the synthetic molecule during their labelling. In addition, it is desirable to reduce the number of steps required for imaging experiments to avoid perturbation of the biological system. Recently, many bioorthogonal, chemistry-based techniques have been developed to study innate biological systems [7,8,9,10]. Owing to the small size, high selectivity, and spatiotemporal controllability of biomolecules, various strategies have been used to label them. These biomolecules include nucleic acids, sugars, lipids, and proteins. In bio-orthogonal chemistry, inverse electron demand Diels–Alder (iEDDA) reactions have recently attracted attention owing to their fast kinetics and excellent specificity [11,12,13]. The iEDDA reaction is based on the interaction between tetrazine [14] and strained olefins (such as norbornene, *trans*-cyclooctene (TCO), and cyclopropane). Regarding the development of a fluorescent probe, one of the most important features of the iEDDA labeling strategy is its potential to impart fluorescence. Tetrazine-functionalized dyes are known to act as fluorogenic probes, where they significantly increase the fluorescence intensity upon their reaction with a strained dienophile such as TCO. These tetrazine-functionalized fluorogenic probes are especially interesting for live-cell labeling and fluorescence imaging applications because the fluorogenic reaction could lower the background noise and potentially eliminate the need to wash off any excess fluorophore. In addition, this feature could be particularly useful for monitoring fast, highly dynamic, biological processes via the in situ fluorescence labeling of cellular compartments that can then be visualized and tracked by various microscopy techniques with spatiotemporal control. Furthermore, the fast kinetics and biocompatibility of tetrazine-functionalized dyes give them great potential for use in fluorescence bioimaging. In this review, we will focus on the syntheses, molecular-design strategies, and bioimaging applications of tetrazine-functionalized fluorogenic probes.

## 2. Synthesis of Tetrazines

As tetrazine-based fluorogenic probes enable a molecular-level understanding of biological systems, synthetic methods have been widely investigated to obtain various types of tetrazine-functionalized fluorophores. There are two distinct approaches for synthesizing tetrazines [13]. Traditionally, tetrazines were obtained via Pinner and “Pinner-like” reactions, which involve condensation and subsequent oxidation reactions with nitriles and hydrazine. Although these conventional approaches have significantly contributed to the synthetic accessibility of tetrazines, they are not applicable to many kinds of tetrazines. Recently, metal-catalyzed C–C bond formation using convenient tetrazine intermediates has received significant attention as an alternative method. In this section, we discuss recent findings that can successfully improve the utility and scope of the reaction. Finally, examples of the synthesis of tetrazine-conjugated fluorophores, which can act as bioprobes, using recently developed methods are presented.

### 2.1. Typical Pinner and “Pinner-Like” Reactions

**Scheme 1:**
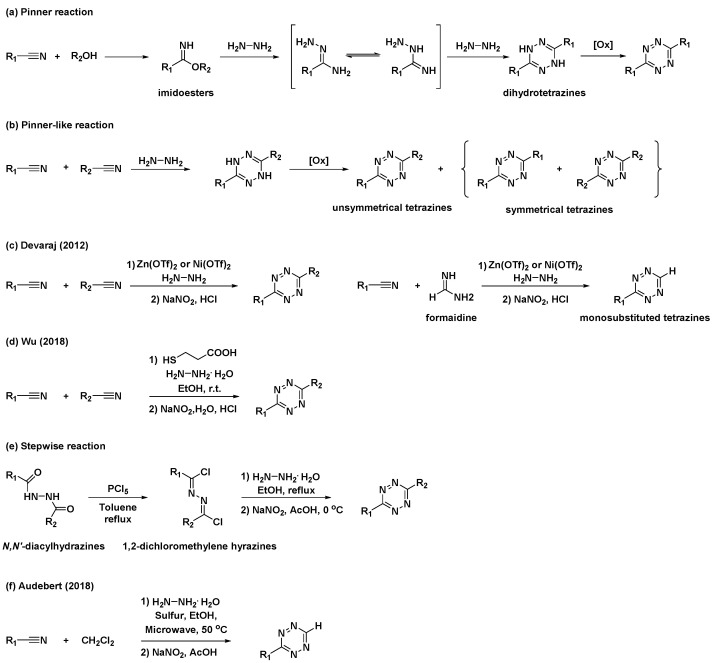
Synthetic approaches to tetrazines through classical Pinner and “Pinner-like” reactions. (**a**) Pinner reaction. (**b**) “Pinner-like reaction. (**c**) Metal-catalyzed Pinner-like reaction. (**d**) Thiol-catalyzed “Pinner-like” reactions. (**e**) Stepwise reaction for preparation of unsymmetrical tetrazines. (**f**) Sulfur-mediated “Pinner-like” reactions.

The first synthetic route to tetrazine, reported by Adolf Pinner, involves the condensation reaction of imidoesters with hydrazine to afford dihydrotetrazine intermediates (Scheme 1a) [15]. The final tetrazines were prepared by the subsequent oxidation of the dihydrotetrazine intermediates. Based on this study, alternative, one-pot syntheses of tetrazines using nitriles and hydrazine (Scheme 1b), commonly known as “Pinner-like” reactions, were reported [16]. Although beneficial, the practical applications of these approaches are restricted because the preparation of unsymmetrical 3,6-disubstituted tetrazines using two different nitriles results in the formation of inevitable byproducts such as symmetrical tetrazines. Moreover, they are not suitable for the synthesis of alkyl tetrazines from aliphatic nitriles [13]. To overcome these limitations, Devaraj et al. developed a metal-catalyzed reaction for the synthesis of a series of alkyl tetrazines, where Zn(OTf)_2_ and Ni(OTf)_2_ act as Lewis acids to promote the addition of hydrazine to inactivate alkyl nitriles (Scheme 1c) [17]. This approach has significantly improved access to alkyl and unsymmetrical tetrazines. Additionally, monosubstituted, unsymmetrical tetrazines can be synthesized in high yields via the condensation of a formamidine salt and nitrile in the presence of a Ni or Zn catalyst. However, this method is limited by the use of reactive and hazardous anhydrous hydrazine. Hydrazine is a good reductant and a good nucleophile; thus, the reactions it is employed in usually offer poor substrate scopes and harsh conditions [14]. To overcome these limitations, organocatalytic approaches for the preparation of unsymmetrical tetrazines were developed by Wu et al. in 2019 (Scheme 1d) [18]. It was suggested that a thiol-containing organocatalyst could promote the formation of unsymmetrical tetrazines at room temperature from hydrazine hydrate and nitriles bearing reactive functional groups. These mild reaction conditions expanded the access to gram-scale synthesis and imparted a broad substrate scope.

Additionally, several studies have been conducted to address unmet needs. For example, applications of *N,N*′-diacylhydrazines for the synthesis of unsymmetrical tetrazines have been reported (Scheme 1e) [19]. *N,N*′-Diacylhydrazines were prepared by the sequential introduction of acyl groups into hydrazine. They were first converted to 1,2-dichloromethylene hydrazines by treatment with PCl_5_, followed by the condensation of hydrazine and subsequent oxidation to yield unsymmetrical tetrazines with aliphatic substituents. Sulfur-mediated “Pinner-like” reactions generating reactive nucleophile NH_2_NHSH were generally used to synthesize aromatic tetrazines [20]. Recently, Audebert et al. demonstrated that dichloromethane (DCM) could be used as an alternative reagent for the formamidine salt when preparing monosubstituted unsymmetrical tetrazines via sulfur-mediated “Pinner-like” reactions (Scheme 1f) [21].

### 2.2. Tetrazine Synthesis Based on C–C Bond Formation

**Scheme 2:**
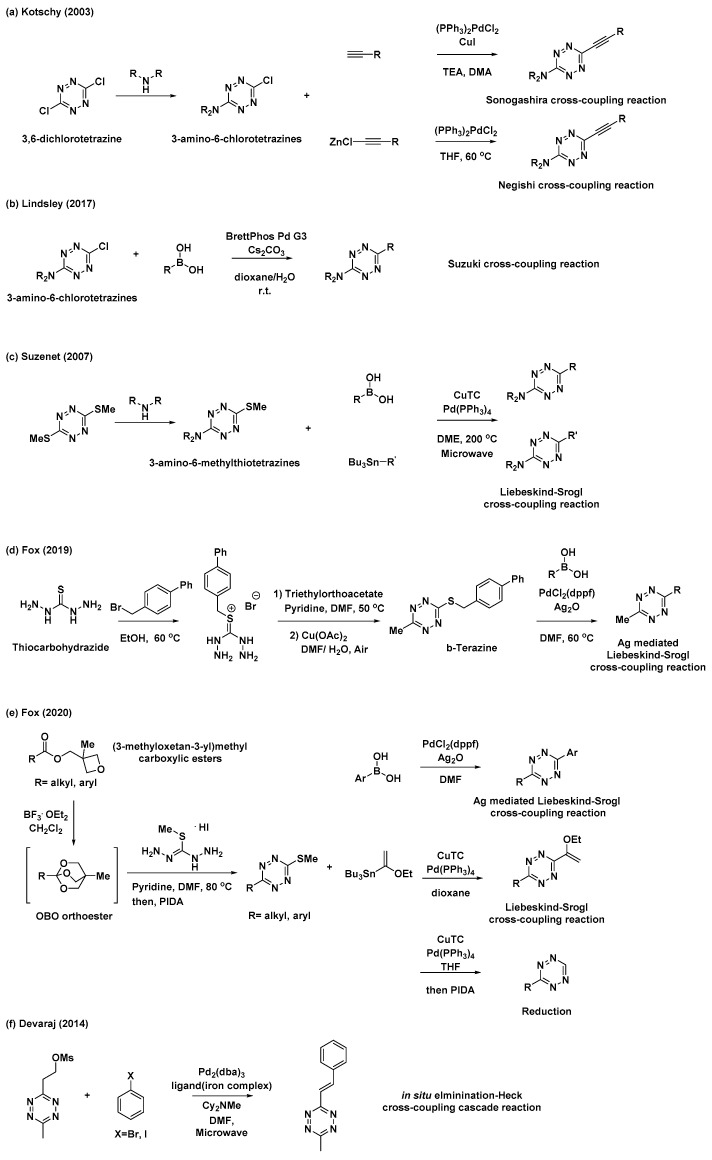
Synthetic approaches to tetrazines through metal-catalyzed C–C bond formations. (**a**) Sonogashira or Negishi cross-coupling reaction of chlorotetrazine. (**b**) Suzuki cross-coupling reaction of chlorotetrazine. (**c**) Liebeskind–Srogl cross-coupling reaction of thioether tetrazines. (**d**) Ag-mediated Liebeskind–Srogl cross-coupling reaction of b-Tetrazine. (**e**) Liebeskind–Srogl cross-coupling reaction or reduction of 3-substituted-6-thiomethyltetrazines. (**f**) Elimination/Heck cross-coupling cascade reaction.

Despite notable advances in “Pinner-like” reactions, it is challenging to develop synthetic methods for unsymmetrical 3,6-disubstituted tetrazines with a broad substrate scope and high utility. While previous studies mostly focused on the synthesis of tetrazines through the condensation of nitriles and hydrazine, the feasibility of metal-catalyzed C–C bond formation reactions has rendered versatile tetrazine intermediates as attractive targets for the synthesis of unsymmetrical disubstituted tetrazines. Kotschy et al. reported a new method for the preparation of alkynyl tetrazines through Sonogashira or Negishi cross-coupling reactions with chlorotetrazine intermediates (Scheme 2a) [22]. The coupling partners, 3-amino-6-chlorotetrazine intermediates, were synthesized by nucleophilic aromatic substitution, where one chlorine atom of 3,6-dichlorotetrazine was replaced with different amine nucleophiles. Although the substrate scope of tetrazine intermediates is limited due to decomposition, this method has significant potential for the synthesis of new tetrazines via C–C bond formation. As a result, significant efforts have been devoted to the application of other metal-catalyzed cross-coupling reactions and the development of tetrazine intermediates. Recently, Lindsley et al. reported the use of a Suzuki cross-coupling reaction to introduce aryl, heteroaryl, and vinyl groups onto 3-amino-6-chlorotetrazines (Scheme 2b) [23].

Thioether tetrazines are one of the most useful intermediates for the synthesis of unsymmetrical disubstituted tetrazines. Suzenet et al. reported the Liebeskind–Srogl cross-coupling reaction of disubstituted thioether tetrazines with various boronic acids and organostannanes to yield tetrazines with aryl, heteroaryl, and vinyl substituents (Scheme 2c) [24]. Similar to 3-amino-6-chlorotetrazines, 3-amino-6-methylthiotetrazines were easily obtained from 3,6-bis(methylthio)-1,2,4,5-tetrazine through nucleophilic displacement. This approach has sparked great interest in the development of other valuable thioether tetrazine intermediates. In 2019, Fox et al. demonstrated that 3-thioalkyl-6-methyltetrazines, especially 3-((*p*-biphenyl-4-ylmethyl)thio)-6-methyltetrazine (b-tetrazine), could serve as a useful intermediate for the synthesis of 3-aryl-6-methylterazines through Ag-mediated Liebeskind–Srogl cross-coupling reactions (Scheme 2d) [25]. To prepare b-tetrazine on a decagram scale, thiocarbohydrazide was subjected to S-alkylation with 4-bromomethylbiphenyl, followed by the condensation of the resultant salt with triethylorthoacetate and subsequent Cu(OAc)_2_-catalyzed air oxidation. Compared to those of the typical, copper(I)-mediated Liebeskind–Srogl cross-coupling reaction, the synthetic scope and utility of this Ag-mediated reaction were significantly broader. Furthermore, this new Liebeskind–Srogl cross-coupling protocol is an outstanding synthetic method for the preparation of unsymmetrical tetrazines. In 2020, the same group also synthesized 3-substituted-6-thiomethyltetrazines intermediates bearing various alkyl or aryl groups (Scheme 2e) [26]. This versatile synthetic route begins with the conversion of (3-methyloxetan-3-yl)methyl carboxylic esters into oxabicyclo [2.2.2]octyl (OBO) orthoester intermediates in the presence of BF_3_∙OEt_2_. These compounds then undergo further condensation reactions with *S*-methylisothiocarbohydrazide hydroiodide and subsequent oxidation to afford the 3-substituted-6-thiomethyltetrazines intermediates. These intermediates can be converted into unsymmetrical tetrazines through the Liebeskind–Srogl cross-coupling reaction, as well as into monosubstituted tetrazines through catalytic thioether reduction. It is worth mentioning that these synthetic methods provide convenient access to various unsymmetrical, aliphatic, aromatic, and heterocycle-substituted tetrazines.

Furthermore, Devaraj et al. prepared a novel mesylate tetrazine precursor that could react with aryl halides through cascades of eliminations and Heck cross-couplings reaction, affording various (*E*)-3-substituted-6-alkenyltetrazines (Scheme 2f) [27]. Using this method, tetrazine derivatives with different π-conjugation lengths can be easily prepared. 

### 2.3. Conjugation of Tetrazine to Fluorophores

**Scheme 3:**
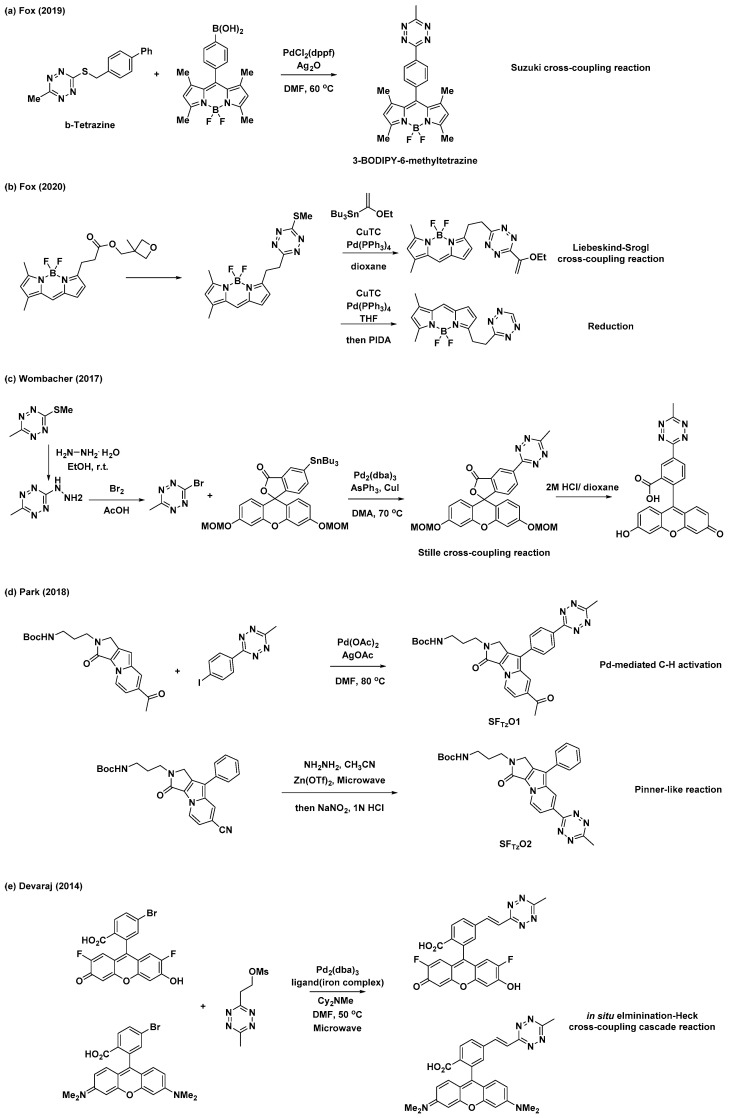
Synthetic examples of tetrazines conjugated with various fluorophores. (**a**) Ag-mediated Liebeskind–Srogl cross-coupling reaction for modification of BODIPY core. (**b**) Liebeskind–Srogl cross-coupling reaction or reduction for synthesis of a BODIPY dye with mono-or di-substituted tetrazine. (**c**) Stille cross-coupling reaction for preparation of 3-fluorescein-6-methyltetrazine. (**d**) Pd-mediated C–H activation or Zn-catalyzed “Pinner-like” reaction for synthesis of Seoul-Fluor (SF) scaffold with tetrazine. (**e**) Elimination/Heck cross-coupling cascade reaction for preparation of Oregon-Green and tetramethylrhodamine (TMR) derivatives with π-conjugated tetrazine.

In addition to the synthetic endeavors to address the challenges associated with tetrazine, various studies have been conducted on the conjugation of tetrazine to fluorophores for applications in the field of bioorthogonal chemistry. As mentioned previously, 3-thioalkyltetrazine can be converted into various unsymmetrical 3,6-disubstituted tetrazines. Thus, Fox et al. synthesized 3-BODIPY-6-methyltetrazine (BODIPY = 4,4-difluoro-4-bora-3a,4a-diaza-s-indacene) modifying a BODIPY core with b-tetrazine and phenyl boronic acid through an Ag-mediated Liebeskind–Srogl coupling reaction (Scheme 3a) [25]. Additionally, they demonstrated the application of (3-methyloxetan-3-yl)methyl carboxylic esters with BODIPY for the synthesis of a BODIPY dye with mono-or di-substituted tetrazines (Scheme 3b) [26]. Wombacher et al. utilized Stille cross-coupling to prepare 3-fluorescein-6-methyltetrazine (Scheme 3c) [28]. They prepared another tetrazine intermediate, 3-bromo-6-methyltetrazine, from 3-(methylthio)-6-methyltetrazine through nucleophilic addition of hydrazine followed by bromination. Park et al. utilized Pd-mediated C–H activation for the incorporation of the tetrazine moiety into the Seoul-Fluor (SF) scaffold to synthesize SF_Tz_01 (Scheme 3d) [29]. SF_Tz_02 could also be synthesized through a Zn-catalyzed “Pinner-like” reaction (Scheme 3d). In addition, Oregon-Green and tetramethylrhodamine (TMR) derivatives with π-conjugated tetrazines were prepared via cascades of eliminations and Heck cross-couplings reaction using a mesylate tetrazine precursor (Scheme 3e) [27].

## 3. Development of Fluorogenic Tetrazine Fluorophores

Tetrazine has been used as a fluorescence quencher. Depending on the design strategy, tetrazine can non-radiatively release molecular energy via different mechanisms, thereby quenching the fluorescence. After the iEDDA reaction, pathways for the non-radiative release of energy are no longer available; therefore, fluorescence is restored. Consequently, fluorophores conjugated with tetrazine could be used as bioorthogonal fluorogenic probes for wash-free molecular imaging. Fluorophores modified by various tetrazines and four different types of quenching mechanisms have been used to develop tetrazine-modified fluorophores. These include electronic energy transfer (EET) (e.g., Förster resonance energy transfer (FRET), Dexter, and through-bond energy transfer (TBET)), monochromophoric design, and the formation of new fluorophores after an iEDDA reaction. EET-type fluorogenic probes typically employ the energy of an excited molecule. FRET is one type of EET that takes place between the excited states of donor molecules and the ground states of acceptor molecules via non-radiative dipole–dipole interactions. For this process, the distance between the donor and acceptor is important, and the emission spectrum of the donor must overlap with the absorption of the acceptor. Due to the uniqueness of this process, there are many applications employing FRET phenomena for the development of molecular sensors and investigations into bioimaging probes [30]. In contrast to FRET, Dexter-type EET [31] does not require spectral overlap between the donor and acceptor molecules. Instead, electron hopping occurs from the donor to the acceptor molecule; therefore, the wavefunctions of both the donor and acceptor must overlap. In general, Dexter-type EET requires a shorter distance between the donor and acceptor molecules than that required by FRET-type EET. TBET [32,33] is the EET between a donor and acceptor that occurs through an electronically conjugated, rigid π-system linker, which twists the donor and acceptor groups out of co-planarity. It is still difficult to explain the exact mechanism of TBET or to differentiate TBET from FRET; however, the fast energy transfer rate of TBET, which can be suppressed at low temperatures, is a key characteristic of this process that could be used to examine the mechanism behind its occurrence. The basic principle of EET-type strategies is based on the non-radiative decay of fluorophore energy, where the energy is transferred to a fluorescence quencher (e.g., tetrazine) from the fluorophore; thus, it is important to optimize the efficiency of this energy transfer. In contrast to EET-type quenching, the monochromophoric-design strategy is based on the dark-state quenching of tetrazine. Before the iEDDA reaction, the optically forbidden n→π* transition dominates the S_0_–S_1_ transition of the molecule, which becomes a π→π* transition after the reaction. The main advantage of the monochromophoric-design strategy is that the unique mechanism it employs allows for the development of a wider range of fluorogenic probes than that offered by EET-type quenching strategies. On the other hand, several studies have reported the formation of new fluorophores after an iEDDA reaction. Because there is no fluorescent molecule before the reaction, this strategy provides a high turn-on ratio. In the subsequent Section 3.1, Section 3.2, Section 3.3, Section 3.4 and Section 3.5, we present an example of each strategy and the biological applications of the resultant probes.

### 3.1. FRET-Type EET Strategy

**Figure 1:**
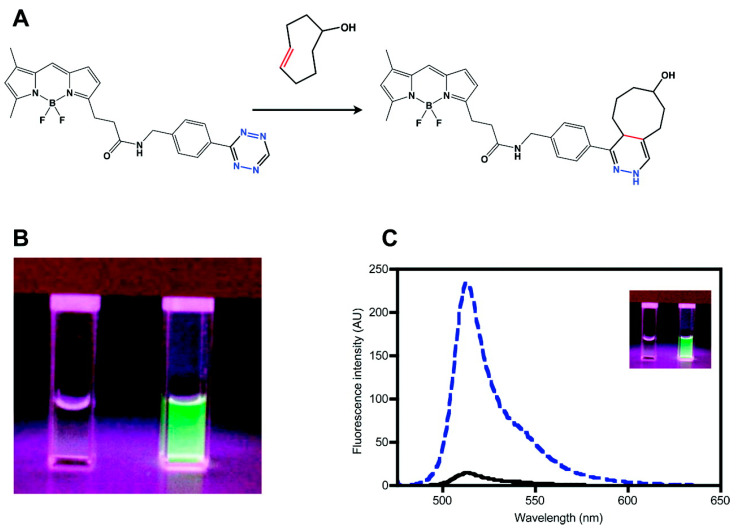
Synthesis of a tetrazine-functionalized fluorophore via Förster resonance energy transfer (FRET). (**a**) Schematic representation of the reaction between *trans*-cyclooctenol and tetrazine-BODIPY-FL (BODIPY = 4,4-difluoro-4-bora-3a,4a-diaza-s-indacene) via an inverse electron demand Diels–Alder (iEDDA) reaction. (**b**) and (**c**) Fluorogenic properties of tetrazine–BODIPY-FL. Comparison of the image (**b**) and emission spectrum (**c**) of tetrazine–BODIPY-FL (left cuvette and black line, respectively) and the corresponding dihydropyrazine product (right cuvette and dashed blue line, respectively). Reproduced with permission from [36]. Copyright (2018) American Chemical Society.

The most convenient way to modify a fluorophore is to conjugate the fluorophore by amide coupling with the amine form of tetrazine [34]. This approach is based on FRET-type fluorescence quenching, wherein energy is transferred from the excited fluorophore to the tetrazine units. The transferred energy is released by the tetrazine in a non-radiative manner. The first example of the fluorescence quenching effect of tetrazine was reported by Weissleder et al. in 2010 [35]. They conjugated 3-*H*-6-phenyl-1,2,4,5-tetrazine (H-Tet) derivatives with BODIPY-FL, Oregon Green 488, BODIPY TMR-X, and Vivotag-680 dyes and found a 15- to 20-fold enhancement in fluorescence in Phosphate Buffered Saline (PBS) for green- and red-emitting tetrazine dyes (Figure 1). However, there was no quenching observed for near-IR-emitting dyes with this molecular design.

In this study, the probe was used to visualize tubulin filaments using Taxol^®^–TCO. They confirmed that TCO-modified Taxol^®^ could inhibit tubulin polymerization. In addition, they confirmed that tetrazine–BODIPY-FL could be successfully used to visualize microtubule bundles with Taxol^®^–TCO. Although H-Tet was used earlier for the development of fluorogenic tetrazine-functionalized fluorophores, the majority of the commercially available tetrazine-functionalized fluorophores exhibiting FRET behavior are conjugated with 3-methyl-6-phenyl-1,2,4,5-tetrazine (Me-Tet) derivatives. Since tetrazine acts as the lowest occupied molecular orbital (LUMO) for the iEDDA reaction, an increase in the electron-withdrawing character at the 3-position of tetrazine would promote the reaction rate by lowering the LUMO energy level. Enhancing the reactivity of tetrazine by increasing the electron-withdrawing character makes them susceptible to nucleophilic attack. Therefore, the most prominent differences between Me-Tet and H-Tet for fluorescence imaging are the reaction kinetics and chemical stability. Consequently, H-Tet has faster reaction kinetics but lower chemical stability than those of Me-Tet.

### 3.2. Dexter-Type, Electron-Exchange EET Strategy

**Figure 2:**
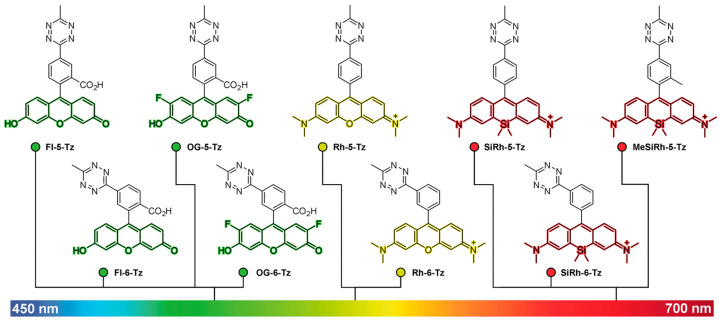
Tetrazine-functionalized fluorophore via a Dexter-type, electron-exchange strategy. This figure represents the chemical structures and emission wavelengths of the green to far-red emissions of fluorogenic tetrazine-functionalized xanthene derivatives. Reproduced from [28] with permission from the Royal Society of Chemistry.

Because the FRET-based strategies for fluorescence quenching have higher efficiency with regard to the spectral overlap of the donor and acceptor fluorophores, their quenching efficiency is wavelength-dependent. Since tetrazine has an absorption maximum of approximately 530 nm, the quenching efficiency of tetrazine decreases with fluorophores exhibiting a red shift. To overcome this limitation, Dexter-type electron exchange strategies have been examined, which involve the direct incorporation of tetrazines into fluorophores to decrease the distance between the donor (fluorophore) and acceptor (tetrazine) molecules. For instance, Wombacher et al. reported green- to far-red-emitting fluorogenic tetrazine probes, which were prepared by the direct conjugation of tetrazine with xanthene fluorophores via C–C bond-forming reactions [28]. As this molecular design provides a short distance between tetrazine and the fluorophore, this strategy allowed for the development of xanthene–tetrazine derivatives that exhibited fluorescence over the broad visible range of emission, from green to far-red (Figure 2). This could not be achieved with previous FRET-type molecular-design strategies.

Two- to four-fold fluorescence enhancement, with a far-red emission wavelength (~660 nm), was achieved with silicon rhodamine [37,38] derivatives (SiRh) in this study.

**Figure 3:**
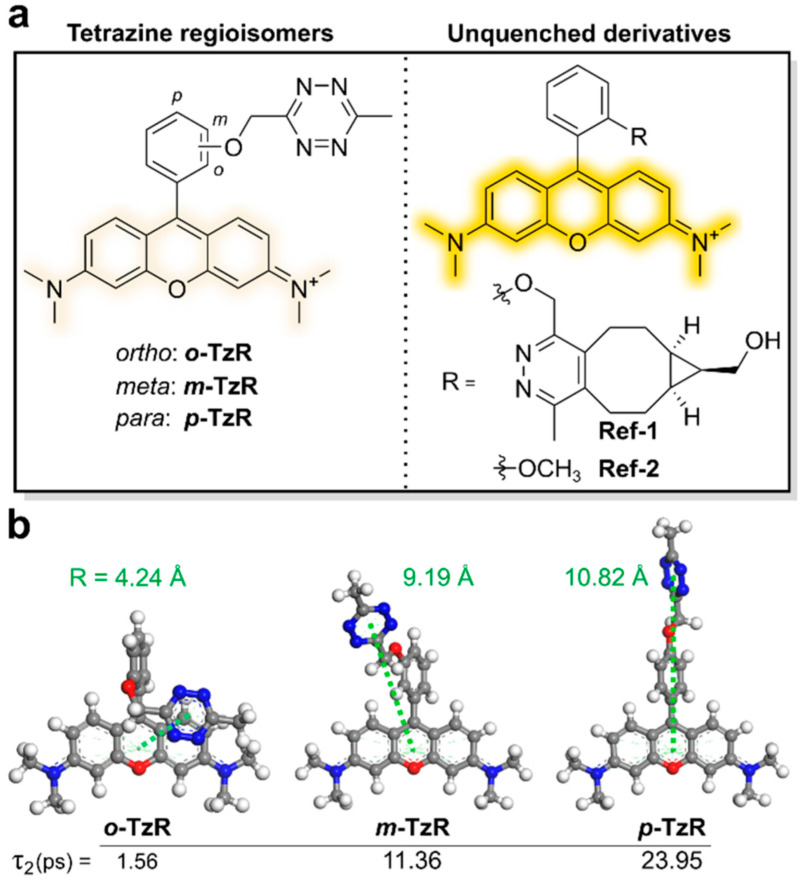
Molecular-design strategy for Dexter-type fluorogenic tetrazine probe. (**a**) Chemical structures of tetrazine isomers and unquenched derivatives of rhodamine dyes. (**b**) Density functional theory (DFT)-optimized structures of regioisomers and expected intermolecular distances between their tetrazine units and xanthene core. Reproduced from [39].

**Figure 4:**
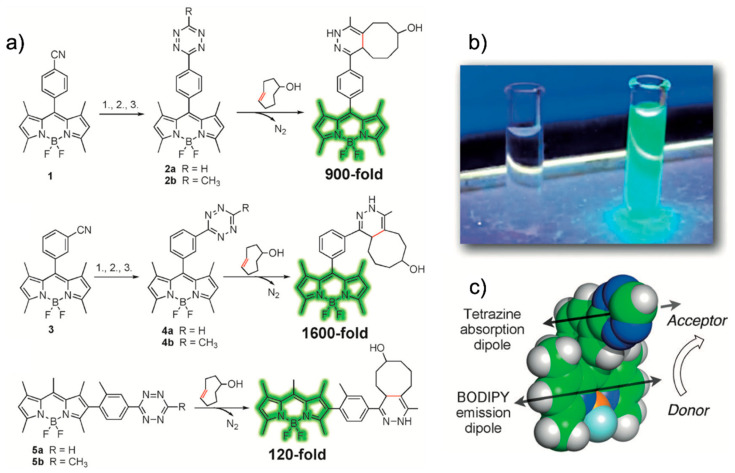
Synthesis of a tetrazine-functionalized fluorophore via a through-bond energy transfer (TBET) strategy. (**a**) Synthetic scheme of BODIPY-*p*-tetrazine derivatives and schematic representation of the fluorogenic reaction with *trans*-cyclooctene (TCO). (**b**) Photographs of BODIPY-tetrazine probes without (left) and with (right) TCO, upon excitation under a handheld UV lamp. (**c**) Schematic representation of the orientation of the donor (BODIPY core) and acceptor (tetrazine) transition dipoles. Reproduced from [41] with permission from Wiley-VCH.

Recently, Wombacher et al. reported some cell-permeable and impermeable dyes, including highly fluorogenic far-red emitting derivatives [39]. To increase the fluorescence quenching efficiency, they synthesized tetrazine-functionalized fluorophores using TMR and SiRh with a phenyl ring pendent in the xanthene core having different tetrazine positions (*ortho*, *meta*, or *para*) with short flexible oxymethyl spacers (Figure 3a). Density functional theory (DFT) calculations suggested that the regioisomers with tetrazine connected at different positions have different proximities between the fluorophore and tetrazine, resulting in better quenching efficiency (Figure 3b). Dexter-type electron exchange was confirmed by recording the femtosecond transient absorption spectra. *ortho*-Tetrazine substituents provided higher fluorescence quenching efficiency than those of *meta*- or *para*-tetrazine substituents, with a 95-fold increase in fluorescence turn-on. In this study, HD653 (em = 676 nm) exhibited a 50-fold increase in fluorescence turn-on. Furthermore, the probe was used for multicolor, no-wash imaging of the target-of-interest (TOI) protein. By modifying the chemical structure, the cell permeability of this tetrazine probe was controlled and it was successfully used for fluorogenic bioorthogonal imaging of the TOI protein labeled with Bicyclononyne-containing, unnatural amino acids (Figure 4). In addition, the self-blinking tetrazine probe allowed for the stimulated emission depletion (STED) imaging of the TOI protein and organelles, including UAA-labeled vimentin, H2A protein, mitochondria, and actin filaments [39,40].

### 3.3. TBET-Type EET Strategy

Weissleder et al. reported a fluorogenic tetrazine-functionalized fluorophore based on the TBET mechanism [41]. By directly conjugating tetrazine with BODIPY, they achieved a highly fluorogenic probe whose fluorescence turn-on magnitude was on the order of 10^3^. These super-dark probes are important for the fluorescence imaging of less abundant proteins or for super-resolution imaging [5]. They also synthesized tetrazine-functionalized BODIPY derivatives (Figure 4a) to enhance the spatial donor–acceptor proximity and to provide predictable donor–acceptor transition-dipole orientations, which could enable access to alternative modes of fluorescence quenching. In this study, the conjugation of a phenyl pendent to a tetramethyl BODIPY fluorophore exhibited 1600-fold fluorescence enhancement after an iEDDA reaction (Figure 4a,b). The authors claimed that their molecular design resulted in a parallel alignment of the tetrazine absorption dipole and BODIPY emission dipole, thereby maximizing the TBET (Figure 4c). Using phalloidin-TCO, they successfully visualized actin filaments via fluorescence with a high signal-to-noise (S/N) ratio.

**Figure 5:**
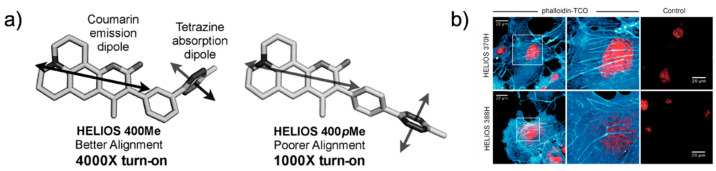
Coumarin-tetrazine probes for fluorogenic imaging. (**a**) Schematic representation of the orientation of the transition dipole between coumarin and tetrazine. (**b**) No-wash, bio-orthogonal fluorogenic imaging of actin filament with phalloidin-TCO. COS-1 cells were sequentially incubated with phalloidin-TCO and DRAQ5. After brief rinsing, cells were imaged upon the addition of the coumarin–tetrazine probe. Reproduced from [42] with permission from Wiley-VCH.

Soon after, Weissleder et al. reported a fluorogenic bioorthogonal probe based on a coumarin fluorophore [42]. Employing their TBET strategy, they modified the coumarin fluorophore to synthesize hyperemissive ligation-initiated orthogonal sensing (HELIOS) probes having a phenyl pendent with tetrazine at the *meta*-position of the coumarin core (Figure 5). Depending on the coumarin structure, the HELIOS probe can have a varying blue-green spectrum (from 455 to 502 nm) with a high turn-on ratio (2500- to 11,000-fold enhancement). This study again highlighted that the transition dipole between the fluorophore and tetrazine is important for efficient fluorescence quenching (Figure 5a).

The HELIOS probe was used to visualize the actin cytoskeleton to investigate the imaging potential of the probe. By sequential addition of phalloidin-TCO and HELIOS probes, vivid fluorogenic images of the actin filament with excellent S/N ratios were successfully obtained (Figure 5b).

### 3.4. Monochromophoric Design

**Figure 6:**
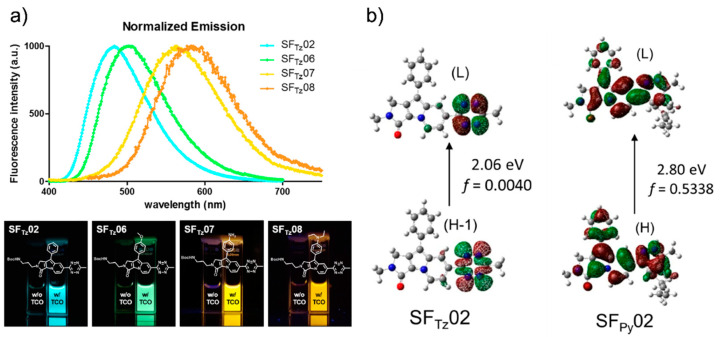
Development of a monochromophore-based, tetrazine-functionalized fluorophore. (**a**) Upper panel: Normalized emission spectrum of SF_Tz_ (SF = Seoul-Fluor) probes after reaction with TCO. Lower panel: Photograph of the SF_Tz_ probes before (left cuvette) and after (right cuvette) reaction with TCO; irradiated under 365-nm UV light. (**b**) Molecular orbital distribution, vertical transition energy, and oscillator strength (*f*) of SF_Tz_02 (left) and the corresponding iEDDA reaction product (right) obtained by Time-Dependent-DFT calculations (CAM-B3LYP/6-31G*) of the corresponding first excited-state optimized structures. Reproduced with permission from [29]. Copyright (2018) American Chemical Society.

Recently, a dark-state quenching phenomenon has been reported. This is based on the monochromophoric design strategy, which involves a coupled, conjugated π system between the fluorophore and tetrazine in the same plane. This strategy is not based on donor-to-acceptor energy transfer or on the general design strategy for biochromophores. Moreover, the quenching efficiency of the system was preserved regardless of the emission wavelength of the parent fluorophores. For example, Park et al. reported a new molecular-design approach, which provided a 600- to 1000-fold increase in turn-on efficiency for blue to red emission (Figure 6a).

**Figure 7:**
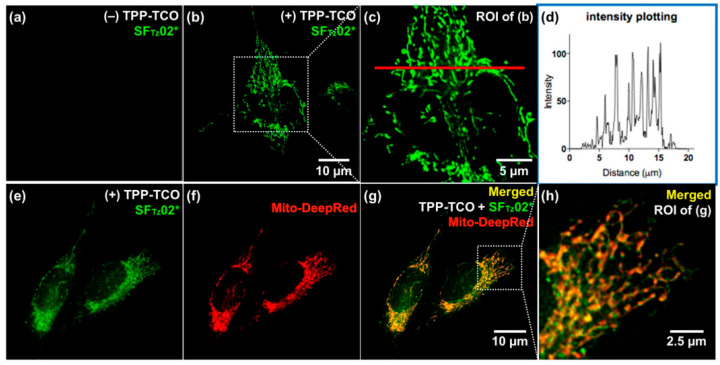
No-wash fluorogenic imaging of mitochondria with SF_Tz_ probes. HeLa cells were treated with dimethylsulfoxide (**a**) or triphenylphosphonium (TPP)−TCO (10 μM; **b**, **c**, **e**, **g**, and **h**) with MitoTracker Deep Red (**f**−**h**). After brief washing with phosphate buffered saline, SF_Tz_02* (**a**, **b**, **c**, **e**, **g**, and **h**) was used to treat the cells, and the images were immediately observed without washing. (**d**) Plot intensity values of pixels along the red line in (**c**) were analyzed with the ImageJ program. ROI = region of interest. Reproduced with permission from [29]. Copyright (2018) American Chemical Society.

They confirmed full integration between tetrazine and a model, SF fluorophore system [43] by acquiring the absorption spectra. In addition, they found that the oscillator strength (*f*) of the structure significantly increased after the iEDDA reaction. Based on this observation, they proposed that the iEDDA reaction changed the major contributor of the S_0_–S_1_ transition of the probes from the optically forbidden n→π* transition to the optically active π→π* transition, thereby promoting the fluorescence emission process (Figure 6b). Considering the increased interest in mitochondrial biology, a new tetrazine fluorophore was used to visualize the mitochondria (Figure 7). Triphenylphosphonium (TPP)–TCO was synthesized and incubated with HeLa cells. After treatment with SF_Tz_02* or SF_Tz_08* (the more soluble forms of SF_Tz_02 and SF_Tz_08, respectively), crisp fluorescent mitochondrial images with exceptional contrast were obtained. They further confirmed the specificity of the probe through a co-staining experiment with a conventional fluorescent mitochondrial probe, MitoTracker Deep Red.

### 3.5. Formation of New Fluorophore

**Figure 8:**
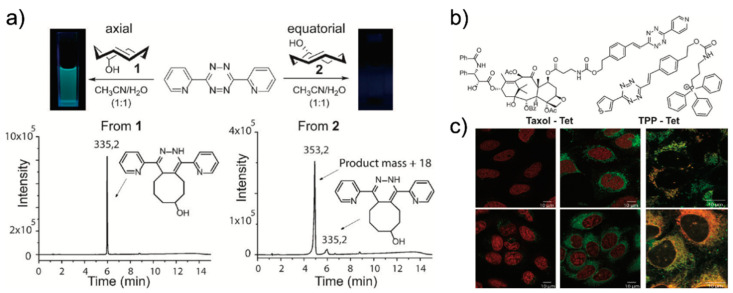
Formation of a new fluorophore after an iEDDA reaction. (**a**) Reaction between 3,6-di(2′-pyridyl)-5-tetrazine (DiPyTet) and TCO gave different products. In this study, the reaction of the axial TCO isomer (TCOa) with DiPyTet resulted in new fluorophore formation. (**b**) Chemical structure of tetrazine modified with Taxol^®^ and TPP (Taxol^®^–Tet and TPP–Tet, respectively) in this study. (**c**) Confocal laser microscope images of U2OS cells treated with Taxol^®^–Tet (upper row) or TPP–Tet (lower row), followed by DMSO (left column) or TCOa (middle column) treatment. The merged images obtained from co-staining experiments with commercial mitotracker or tublin tracker (right column) confirmed the specificity of the probes. Reproduced from [44].

Owing to their structure, tetrazines are intrinsically unstable under biological conditions. Therefore, this instability could restore the fluorescence signal of a tetrazine-modified fluorophore without the iEDDA reaction. To address this, Vrabel et al. reported a new concept involving the formation of fluorescent products upon the iEDDA reaction between tetrazine and a particular TCO (Figure 8) [44]. It was found that the reaction between 3,6-di(2′-pyridyl)-5-tetrazine (DiPyTet) and the axial TCO isomer (TCOa) generated a fluorescent product. Through a Heck cross-coupling reaction, symmetric and asymmetric tetrazine derivatives bearing various substituents were synthesized. Interestingly, they found that the iEDDA reaction products of tetrazine derivatives and TCOa exhibited varying photophysical properties. The products exhibited a broad range of emissions (478–605 nm) and varying turn-on ratios (9- to 91-fold enhancement). By conjugating TPP and Taxol^®^ to tetrazine (TPP–Tet and Taxol^®^–Tet, respectively), the modified tetrazines were successfully applied to bioorthogonal fluorogenic bioimaging.

Recently, Varbel et al. reported an extended approach of their strategy. Compared to previous studies, wherein TCOa was used for fluorescent product formation, in this study, they developed new tetrazine derivatives that could afford fluorescent products from both TCOa and equatorial-TCO (TCOe) [45]. Similar to their previous study, they synthesized alkenyl tetrazine derivatives with different substituents. Interestingly, many of the tetrazine derivatives afforded fluorescent products after undergoing iEDDA reactions with TCOa, and most of the 4,5-dihydropyridazine products exhibited red-shifted emissions (626 to 643 nm) and reasonable turn-on ratios (3- to 18-fold enhancement). Furthermore, they conjugated tetrazine with TPP and concanavalin A (ConA-TCO) and used the resultant tetrazine derivative for bio-orthogonal fluorogenic imaging.

## 4. Conclusions

Herein, we briefly summarized the synthetic methods and molecular-design strategies for tetrazine-functionalized fluorogenic bioprobes. The high selectivity, exceptional reaction kinetics, and fluorescence-quenching ability of tetrazine render its use in iEDDA reactions as an effective platform for bio-orthogonal and catalyst-free fluorogenic bioimaging. A basic understanding of the quenching mechanism of tetrazine allows for the fine-tuning of tetrazine-functionalized fluorophores for enhanced emission and fluorescence. Considering the super-resolution imaging capabilities of tetrazine-functionalized fluorophores produced via iEDDA reactions, this reaction serves as a molecular tool for fluorescent bioimaging and will provide a better understanding of the dynamic nature of biological systems. Although tetrazine-functionalized fluorophores exhibit promising potential [46,47,48,49], there are several limitations that must be overcome. For instance, (1) the strained olefin still needs to be washed off before treatment with the tetrazine fluorophore, (2) the electrophilic nature of tetrazine renders it unstable (depending on its substituents) against nucleophiles, and (3) the hydrophobic nature of tetrazine increases the possibility of it partaking in non-specific binding. These limitations must be addressed in future studies. 

## References

[1] Dean K., Palmer A. (2014). Advances in fluorescence labeling strategies for dynamic cellular imaging. Nat. Chem. Biol..

[2] Lavis L.D., Raines R.T. (2014). Bright Building Blocks for Chemical Biology. ACS Chem. Biol..

[3] Lavis L.D., Raines R.T. (2008). Bright Ideas for Chemical Biology. ACS Chem. Biol..

[4] Gonçalves M.S.T. (2009). Fluorescent Labeling of Biomolecules with Organic Probes. Chem. Rev..

[5] Fernández-Suárez M., Ting A.Y. (2008). Fluorescent probes for super-resolution imaging in living cells. Nat. Rev. Mol. Cell Biol..

[6] Lavis L.D., Chao T.-Y., Raines R.T. (2006). Fluorogenic Label for Biomolecular Imaging. ACS Chem. Biol..

[7] Sletten E.M., Bertozzi C.R. (2009). Bioorthogonal Chemistry: Fishing for Selectivity in a Sea of Functionality. Angew. Chem. Int. Ed..

[8] Patterson D.M., Nazarova L.A., Prescher J.A. (2014). Finding the Right (Bioorthogonal) Chemistry. ACS Chem. Biol..

[9] Nguyen S.S., Prescher J.A. (2020). Developing bioorthogonal probes to span a spectrum of reactivities. Nat. Rev. Chem..

[10] Devaraj N.K. (2018). The Future of Bioorthogonal Chemistry. ACS Cent. Sci..

[11] Kim E., Koo H. (2019). Biomedical applications of copper-free click chemistry: In vitro, in vivo, and ex vivo. Chem. Sci..

[12] Knall A.-C., Slugovc C. (2013). Inverse electron demand Diels–Alder (iEDDA)-initiated conjugation: A (high) potential click chemistry scheme. Chem. Soc. Rev..

[13] Oliveira B.L., Guo Z., Bernardes G.J.L. (2017). Inverse electron demand Diels–Alder reactions in chemical biology. Chem. Soc. Rev..

[14] Wu H., Devaraj N.K. (2018). Advances in Tetrazine Bioorthogonal Chemistry Driven by the Synthesis of Novel Tetrazines and Dienophiles. Acc. Chem. Res..

[15] Pinner A. (1897). Ueber die Einwirkung von Hydrazin auf Imidoäther. Liebigs Ann. Chem..

[16] Stéen E.J.L., Edem P.E., Nørregaard K., Jørgensen J.T., Shalgunov V., Kjaer A., Herth M.M. (2018). Pretargeting in nuclear imaging and radionuclide therapy: Improving efficacy of theranostics and nanomedicines. Biomaterials.

[17] Yang J., Karver M.R., Li W., Sahu S., Devaraj N.K. (2012). Metal-Catalyzed One-Pot Synthesis of Tetrazines Directly from Aliphatic Nitriles and Hydrazine. Angew. Chem. Int. Ed..

[18] Mao W., Shi W., Li J., Su D., Wang X., Zhang L., Pan L., Wu X., Wu H. (2019). Organocatalytic and Scalable Syntheses of Unsymmetrical 1,2,4,5-Tetrazines by Thiol-Containing Promotors. Angew. Chem. Int. Ed..

[19] Liu D.S., Tangpeerachaikul A., Selvaraj R., Taylor M.T., Fox J.M., Ting A.Y. (2012). Diels–Alder Cycloaddition for Fluorophore Targeting to Specific Proteins inside Living Cells. J. Am. Chem. Soc..

[20] Audebert P., Sadki S., Miomandre F., Clavier G., Vernières M.C., Saoud M., Hapiot P. (2004). Synthesis of new substituted tetrazines: Electrochemical and spectroscopic properties. New J. Chem..

[21] Qu Y., Sauvage F., Clavier G., Miomandre F., Audebert P. (2018). Metal-Free Synthetic Approach to 3-Monosubstituted Unsymmetrical 1,2,4,5-Tetrazines Useful for Bioorthogonal Reactions. Angew. Chem. Int. Ed..

[22] Novák Z., Kotschy A. (2003). First Cross-Coupling Reactions on Tetrazines. Org. Lett..

[23] Bender A.M., Chopko T.C., Bridges T.M., Lindsley C.W. (2017). Preparation of Unsymmetrical 1,2,4,5-Tetrazines via a Mild Suzuki Cross-Coupling Reaction. Org. Lett..

[24] Martin R., Buchwald S.L. (2008). Palladium-Catalyzed Suzuki−Miyaura Cross-Coupling Reactions Employing Dialkylbiaryl Phosphine Ligands. Acc. Chem. Res..

[25] Lambert W.D., Fang Y., Mahapatra S., Huang Z., Ende C.W., Fox J.M. (2019). Installation of Minimal Tetrazines through Silver-Mediated Liebeskind–Srogl Coupling with Arylboronic Acids. J. Am. Chem. Soc..

[26] Xie Y., Fang Y., Huang Z., Tallon A.M., Ende C.W.A., Fox J.M. (2020). Divergent Synthesis of Monosubstituted and Unsymmetrical 3,6-Disubstituted Tetrazines from Carboxylic Ester Precursors. Angew. Chem. Int. Ed..

[27] Wu H., Yang J., Šečkutė J., Devaraj N.K. (2014). In Situ Synthesis of Alkenyl Tetrazines for Highly Fluorogenic Bioorthogonal Live-Cell Imaging Probes. Angew. Chem. Int. Ed..

[28] Wieczorek A., Werther P., Euchner J., Wombacher R. (2017). Green- to far-red-emitting fluorogenic tetrazine probes—Synthetic access and no-wash protein imaging inside living cells. Chem. Sci..

[29] Lee Y., Cho W., Sung J., Kim E., Park S.B. (2017). Monochromophoric Design Strategy for Tetrazine-Based Colorful Bioorthogonal Probes with a Single Fluorescent Core Skeleton. J. Am. Chem. Soc..

[30] Wu L., Huang C., Emery B.P., Sedgwick A.C., Bull S.D., He X.-P., Tian H., Yoon J., Sessler J.L., James T.D. (2020). Förster resonance energy transfer (FRET)-based small-molecule sensors and imaging agents. Chem. Soc. Rev..

[31] Speiser S. (1996). Photophysics and Mechanisms of Intramolecular Electronic Energy Transfer in Bichromophoric Molecular Systems: Solution and Supersonic Jet Studies. Chem. Rev..

[32] Jiao G.-S., Thoresen L.H., Burgess K. (2003). Fluorescent, Through-Bond Energy Transfer Cassettes for Labeling Multiple Biological Molecules in One Experiment. J. Am. Chem. Soc..

[33] Cao D., Zhu L., Liu Z., Lin W. (2020). Through bond energy transfer (TBET)-based fluorescent chemosensors. J. Photochem. Photobiol. C Photochem. Rev..

[34] Beliu G., Kurz A.J., Kuhlemann A.C., Behringer-Pliess L., Meub M., Wolf N., Seibel J., Shi Z.-D., Schnermann M., Grimm J.B. (2019). Bioorthogonal labeling with tetrazine-dyes for super-resolution microscopy. Commun. Biol..

[35] Devaraj N.K., Hilderbrand S., Upadhyay R., Mazitschek R., Weissleder R. (2010). Bioorthogonal Turn-On Probes for Imaging Small Molecules inside Living Cells. Angew. Chem. Int. Ed..

[36] Devaraj N.K., Weissleder R. (2011). Biomedical Applications of Tetrazine Cycloadditions. Acc. Chem. Res..

[37] Lukinavičius G., Umezawa K., Olivier N., Honigmann A., Yang G., Plass T., Mueller V., Reymond L., Corrêa I.R., Luo Z.-G. (2013). A near-infrared fluorophore for live-cell super-resolution microscopy of cellular proteins. Nat. Chem..

[38] Lukinavičius G., Reymond L., Umezawa K., Sallin O., D’Este E., Göttfert F., Ta H., Hell S.W., Urano Y., Johnsson K. (2016). Fluorogenic Probes for Multicolor Imaging in Living Cells. J. Am. Chem. Soc..

[39] Werther P., Yserentant K., Braun F., Grussmayer K., Navikas V., Yu M., Zhang Z., Ziegler M.J., Mayer C., Gralak A.J. Bioorthogonal red and far-red fluorogenic probes for wash-free live-cell and super-resolution microscopy. bioRxiv.

[40] Werther P., Yserentant K., Braun F., Kaltwasser N., Popp C., Baalmann M., Herten D., Wombacher R. (2020). Live-Cell Localization Microscopy with a Fluorogenic and Self-Blinking Tetrazine Probe. Angew. Chem. Int. Ed..

[41] Carlson J.C.T., Meimetis L.G., Hilderbrand S.A., Weissleder R. (2013). BODIPY–Tetrazine Derivatives as Superbright Bioorthogonal Turn-on Probes. Angew. Chem. Int. Ed..

[42] Meimetis L.G., Carlson J.C.T., Giedt R.J., Kohler R.H., Weissleder R. (2014). Ultrafluorogenic Coumarin–Tetrazine Probes for Real-Time Biological Imaging. Angew. Chem. Int. Ed..

[43] Kim E., Lee Y., Lee S., Park S.B. (2015). Discovery, Understanding, and Bioapplication of Organic Fluorophore: A Case Study with an Indolizine-Based Novel Fluorophore, Seoul-Fluor. Acc. Chem. Res..

[44] Vázquez A., Dzijak R., Dračínský M., Rampmaier R., Siegl S.J., Vrabel M. (2017). Mechanism-Based Fluorogenic trans-Cyclooctene–Tetrazine Cycloaddition. Angew. Chem. Int. Ed..

[45] Siegl S.J., Galeta J., Dzijak R., Vázquez A., Río-Villanueva M.D., Dračínský M., Vrabel M. (2019). An Extended Approach for the Development of Fluorogenic trans-Cyclooctene–Tetrazine Cycloadditions. ChemBioChem.

[46] Mao W., Tang J., Dai L., He X., Li J., Cai L., Liao P., Jiang R., Zhou J., Wu H. (2021). A General Strategy to Design Highly Fluorogenic Far-Red and Near-Infrared Tetrazine Bioorthogonal Probes. Angew. Chem. Int. Ed..

[47] Knorr G., Kozma E., Herner A., Lemke E.A., Kele P. (2016). New Red-Emitting Tetrazine-Phenoxazine Fluorogenic Labels for Live-Cell Intracellular Bioorthogonal Labeling Schemes. Chem.–Eur. J..

[48] Loredo A., Tang J., Wang L., Wu K.-L., Peng Z., Xiao H. (2020). Tetrazine as a general phototrigger to turn on fluorophores. Chem. Sci..

[49] Mboyi C.D., Vivier D., Daher A., Fleurat-Lessard P., Cattey H., Devillers C.H., Bernhard C., Denat F., Roger J. (2019). Bridge-Clamp Bis(tetrazine)s with [N]8 π-Stacking Interactions and Azido-s-Aryl Tetrazines: Two Classes of Doubly Clickable Tetrazines. Angew. Chem. Int. Ed..

